# Biogenic Amines’ Content in Safe and Quality Food

**DOI:** 10.3390/foods10010100

**Published:** 2021-01-06

**Authors:** Maria Martuscelli, Luigi Esposito, Dino Mastrocola

**Affiliations:** Faculty of Bioscience and Technology for Food, Agriculture and Environment, University of Teramo, Via R. Balzarini 1, 64100 Teramo, Italy; lesposito2@unite.it (L.E.); dmastrocola@unite.it (D.M.)

Biogenic amines (BAs) are low-molecular-weight, nitrogenous compounds (mainly polar bases) coming from the decarboxylation of free amino acids or by amination or transamination of aldehydes and ketones. To our knowledge, BAs are essential for cellular development and growth, are important regulators of several processes such as brain activity, regulation of body temperature, stomach pH, gastric acid secretion, the immune response, and the synthesis of hormones and alkaloids, among others [1]. Decarboxylation of free amino acids represents the primary way of BAs’ obtention. Microorganisms involved in this process are positive to the decarboxylase enzyme, with the pathways that seem to be strain dependent rather than species specific [2]. At any rate, the presence of proteins (amino acids), favorable growing and fermenting conditions, and the possibility of external contaminations during food processing are important factors in BAs’ increase. An important contribution is also given by several pro-technological strains, in particular lactic acid bacteria (LAB) from the genera *Lactobacillus*, *Leuconostoc*, *Lactococcus*, *Enterococcus*, and *Streptococcus,* were recently deeply reviewed as they are high tyramine producers. Del Rio et al. [3] clarified the harmful effect of this amine in boosting histamine toxicity besides being responsible for the so-called “cheese reaction”. Although starters are generally considered secure and good for both food safety and the general health status of the human body, there does not exist any regulation looking at the decarboxylase positivity of bacteria. As a matter of fact, it is challenging to use BAs’ content in food as a unit of measure to establish food safety. Evidence of strict correlations between personal sensitivity and genetical predisposition for BAs’ intoxication was found. In particular, the compromising of the detoxification system was enacted by mono and di-amine oxidase (MAO and DAO) enzymes in the intestinal epithelium that change for every individual. Great attention should be reserved not only to those subjects consuming mono and di-amino oxidase inhibitors (MAOI and DAOI) drugs, as they may become particularly sensitive to BAs’ action, but should also include those experiencing any impairment in the functioning of the small intestine or kidneys and so, even coeliac subjects, people who suffered surgery, or those who are in treatment for cancer and other pathologies [4,5]. The scientific research is giving growing insights into BAs’ presence in all food matrices including fresh fruit and vegetables, pulses, baby foods, alcoholic beverage [6,7,8], and halal foods [9]. This scenario forces scientists to turn their attention to the fact that all the population is at risk for experiencing BAs’ accumulation by their choices in meal composition, food sources, and of course specific sensitivity. This editorial has collected papers giving an interesting outlook on the content of BAs’ in food and a possible strategy to reduce their occurrence, BAs’ role in the promotion of aroma, and the specific capacity of selected bacteria in promoting their accumulation and/or degradation. All these papers actively contribute to creating a more complete frame on the theme keeping constant the fact that the presence of BAs’ in food represents an essential part of food quality and food safety.
